# Neurorehabilitation in upper limb amputation: understanding how neurophysiological changes can affect functional rehabilitation

**DOI:** 10.1186/s12984-017-0256-8

**Published:** 2017-05-22

**Authors:** Lewis A. Wheaton

**Affiliations:** School of Biological Sciences, Georgia Institute of Technology, 555 14th Street, Atlanta, GA 30332-0356 USA

**Keywords:** Amputation, Neuroplasticity, Prosthetics, Motor learning, Limb loss, Upper limb

## Abstract

**Background:**

Significant advances have been made in developing new prosthetic technologies with the goal of restoring function to persons that suffer partial or complete loss of the upper limb. Despite these technological advances, many challenges remain in understanding barriers in patient adoption of technology, and what critical factors should be of focus in prosthetics development from a motor control perspective. This points to a potential opportunity to improve our understanding of amputation using neurophysiology and plasticity, and integrate this knowledge into the development of prosthetics technology in novel ways. Here, argument will be made to include a stronger focus on the neural and behavioral changes that result from amputation, and a better appreciation of the time-scale of changes which may significantly affect device adaptation, functional device utility, and motor learning implemented in rehabilitation environments.

**Conclusion:**

By strengthening our understanding of the neuroscience of amputation, we may improve the ability to couple neurorehabilitation with neuroengineering to support clinician needs in yielding improved outcomes in patients.

## Background

A common concern in neurorehabilitation is understanding how to use neuroscience to shed light on treatment of neurological disease. In many health concerns such as stroke, movement disorders, and cognitive dysfunction the challenge of “how to treat” has been, and continues to be, shaped by focused and relevant neuroscience research. While this is true for large and growing public health concerns, it is also true for other injuries to the nervous system where, by comparison, the rate of injury may be rarer. The rarity of a problem does not relate to the severity of a problem.

As of 2005, of the 1.5 million persons with limb loss (amputees) an estimated 541,000 persons in the United States suffer from some form of upper limb loss, many from trauma, but also public health concerns such as dysvascular disease and cancer [1]. It is projected that by 2020, there will be 2.2 million amputees in the United States. Worldwide estimates are difficult to gather largely due to the variability in why amputation happens and underreporting in the developing world. Amputation does not directly affect the vast majority of people in the United States (or the world), but the impact of amputation is severe and the resources available to amputees are not ideal. For amputees, the use of artificial limbs (prostheses) can become a vital part of their lives. Unfortunately, rejection and non-functional use is high [2]. While futuristic devices are being designed and tested, they are not commonly used by patients, due to the high cost of devices and fiscal policies of coverage and reimbursement for prosthetic technology [3].

Work in Norway focusing on wear patterns amongst a substantial cohort of amputees identifies some concerns [4]. It was found that while amputees that wear prostheses tend to have high wear times (8+ hours per day), they only use their prostheses for an average of ~50% of daily living tasks (which included naturally higher numbers for bimanual tasks). In this case, wear does not necessarily relate to use. This study also showed that perceived usefulness scores for a variety of daily living tasks using the most popular prosthetic control mechanisms (body-powered and myoelectric) are markedly similar. Other studies have suggested that lower-cost body-powered devices are considered sufficient for daily living and just as functional as advanced myoelectric devices [5–7]. These findings are significant policy drivers; many insurers will not authorize reimbursement of myoelectric or other advanced devices unless unique medical necessity exists [8, 9]. Other approaches such as targeted reinnervation or limb transplantation are available [10] but are invasive, necessitate varying degrees of additional medical burden after amputation, and require more investigation of long-term outcomes [11]. Meanwhile, there is a void of neuroscience research that can inform optimal device design, adaptation and rehabilitation based in neuroscience and motor physiology.

Concepts related to rehabilitation methods and technology in upper limb amputation are not well grounded in empirical data, which has caused concerns about clinical implementation [12]. Despite exciting technological advances in prosthetics, we do not really understand the neurophysiology of amputation and how the motor system may learn and adapt to a new residual and prosthetic limb [13]. The processes of motor learning (both learning how to use the residual limb in its modified state and functional use of the prosthesis) and neuroplasticity (changes within the nervous system to the amputation and integration of prostheses) are vital. Numerous studies of the technological advances in upper limb prosthetics have shown great promise to provide some enhancement to motor control. However, there is a less-frequently discussed issue; there is no technology that has shown immediate sensory establishment of a prosthetic device paired with immediate gains in motor control without a practice-based learning/adaptation period. This suggests that regardless of the technological advances to date, motor learning is a fundamental principle of prosthesis integration that must be considered. If it is possible that multiple prosthesis technological approaches utilize similar mechanisms of neural plasticity or adaptation, and result in similar improvement or challenges to functional integration, what is the gain of one technological approach over the other? Can a deeper understanding of physiological mechanisms from neuroscience help sharpen prosthetics technology development?

The goal of this review is to highlight our understanding of, and areas where there is a scarcity of knowledge about, the neurophysiology relevant to upper limb loss with a particular focus on neural adaptation, plasticity and motor learning. By doing so, the aim is to emphasize the unique contributions a stronger neuroscientific understanding of the role that amputation may have on adaptations to the nervous system. Further, how motor learning principles can facilitate stronger understandings of the role that sensory feedback may pay in modifying neural circuits for prosthesis adaptation. This will hopefully emphasize the vital role of technological development rooted in a stronger understanding of the underlying neurophysiology of amputation. This review will first focus on known concepts from sensory loss that show potential relevance to amputation, which will serve as a platform for consideration of central nervous system processes that can be affected by amputation. Many studies that demonstrate neuroplasticity following amputation have strong connections to studies using augmented ascending sensory feedback. This has great value for technological approaches that seek to harness sensation and identifying mechanisms that could affect successful implementation. Finally, discussion will focus on how this plasticity may affect motor learning which can have significant impact both development of advanced prostheses and training-based approaches intended to improve prosthesis adaptation and usage.

### Concepts from neuroplasticity under sensory loss

Overall, detailed human neurophysiological studies in amputation are quite rare. There are several methodologies to study neurophysiology that may relate to amputation. Studies of acute perturbations to sensory loss are one example. Modelling acute functional limb loss can be achieved through a variety of techniques, one approach being ischemic nerve block (INB). The use of a blood pressure cuff tourniquet to induce INB has been well evaluated [14–18]. These studies generally demonstrate that INB causes muscle paralysis and rapid plasticity to the limb undergoing INB. Due to the degradation of somatosensory feedback, motor execution relies more on prediction estimates of ongoing limb behavior to supplement diminished proprioceptive input to the somatosensory cortex [19]. While limb paralysis does result from extended INB, prior to paralysis there is no degradation or elimination of motor representations of the limb as identified by remaining descending drive to the deafferented limb [20, 21]. This suggests that INB likely changes how the central nervous system anticipates and plans for motor control and motor accuracy [22]. Mechanistically, data suggests that the motor cortex contralateral to INB increases in excitability through rapid down regulation of gamma-aminobutyric acid (GABA)-related inhibitory circuits [23].

Important for relevance to amputation and rehabilitation, the effects of limb loss are not limited to the contralateral hemisphere. Werhahn and colleagues revealed increased motor excitability to the limb opposite INB, which could be inhibited by the GABA-A agonist lorazepam [24]. Due to the GABA-ergic modulation, it was proposed that rapid ipsilateral cortical plasticity promotes cortical excitability to the contralateral limb (i.e., limb opposite INB [25–27]). More on this from the perspective of amputation will follow, though it may be important for understanding motor learning in the amputated limb, and the unaffected limb. From a motor control perspective, INB of the right hand caused transient increased grip strength, tactile discrimination and sensibility in the left hand [28]. Tactile spatial acuity and somatosensory processing also increase in the limb contralateral to deafferentation [25].

**Fig. 1:**
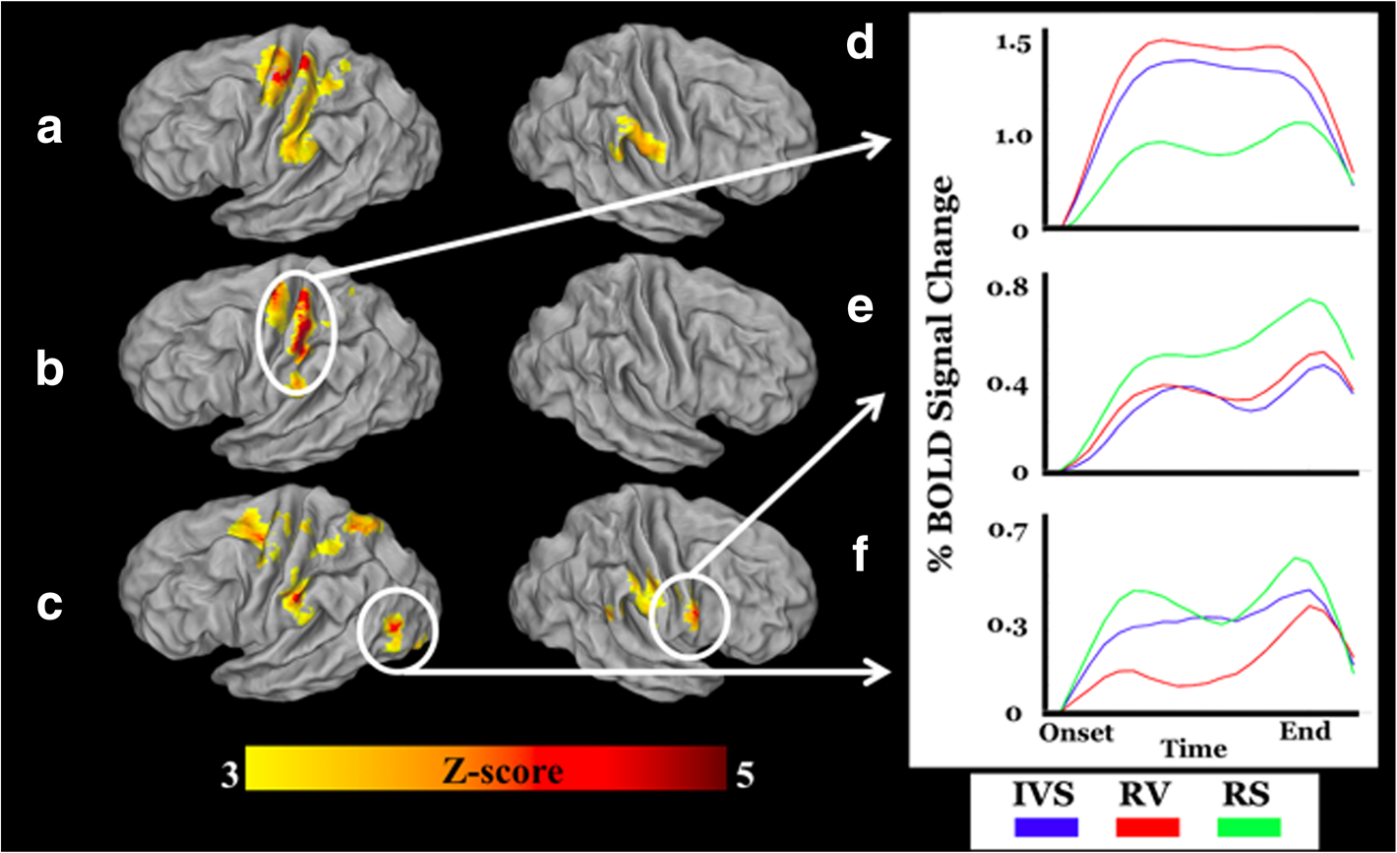
Cortical fMRI activations observed for (**a**) intact sensation, (**b**) reduced visual reliability and (**c**) reduced somatosensory reliability. Time course representations, in percent signal change relative to the mean BOLD response, for three regions of interest: **d** left sensorimotor cortex, **e** left lateral occipital cortex and (**f**) right sensorimotor cortex (from [29], with permission from the publisher)

Using whole-brain neuroimaging techniques, we can illustrate neural changes that suggest plasticity in sensory systems can influence motor control beyond motor cortex. Using functional magnetic resonance imaging (fMRI), we sought to understand the neural systems involved in changing the ability to use visual or somatosensory feedback by selectively reducing the reliability of each sense individually during a tool use task [29]. Participants interacted with a task board to use tweezers to “lift and place” an orange block with the right hand. Participants performed the task with intact visual and proprioceptive sensation, then with reduced vision (with partially occluded glasses), and finally reduced sensation of the right hand with INB. Findings showed that during the task with intact vision and sensation (Fig. 1a), expected left sensorimotor areas were predominantly involved, which increased during reduced vision (Fig. 1b,d). Reduced sensation as a result of INB significantly diminished the activity in left sensorimotor areas and cerebellum, (Fig. 1d) caused increased posterior parietal and occipitotemporal complex activation (Fig. 1f). These areas have been shown to be highly involved in tasks that require hand-eye coordination [30–32], which may be useful in overcoming altered sensorimotor states of the limb that may impair formation of the motor plan. This supports the proposal that lost/impaired proprioception can induce a significant change in sensory weighting for motor control beyond motor cortex [22].

The use of sensation is a vital component for theories of upper limb motor control. Studies have suggested a dual pathway for action based on the forward/inverse model concept [33]. Such models anticipate sensory consequences of upcoming actions. In order for an accurate prediction of the action goal, predicted sensory information is compared to actual sensory information [34, 35]. These sensory-based models have been proposed to be useful for predicting motor outcomes [36]. Relating this to amputation, loss of sensory feedback may perturb the ability to use sensory-based models to function in action prediction and motor planning. In a study done by Moisello and colleagues, 12-h of upper limb immobilization was shown to affect behavioral coordination of limb segments and delayed movement onset, suggesting impaired feedforward control [37]. However, loss of proprioception need not completely impair sensory-based models of motor control, as sensory feedback is also acquired from the visual system [38]. Amputees are capable of interacting with stimuli in peripersonal space, such that their visuospatial representations for motor control are intact [39]. Additionally, previous work has demonstrated that amputees are capable of grip selection planning in their amputated limb, suggestive of the use of internal representations of actions that are shared from the intact limb [40]. Thus, it seems possible that even without bionic prostheses, the motor system may adapt using sensory estimates from alternative sources [19]. Unfortunately, we do not know the scale of neural adaptations that accompany amputation. In line with our previously described fMRI evaluation of sensory weighting [29], recent evidence suggests a putative visual adaptation that may have great relevance to behavior and rehabilitation.

### Neurobehavioral adaptations following amputation

#### Cortical plasticity

Unlike central nervous pathology (such as stroke where a lesion disrupts local and regional brain function), traumatic amputation is thought to not commonly damage the structure of the cerebrum (there is debate about cortical and white matter anatomical changes [41–43] which, if present, appear to better correlate with phantom sensations than motor function [44]). Substantial functional changes have been shown to occur in amputation indicative of neuroplasticity. It is clear that amputation affects motor cortex function by promoting an expansion of the residual limb segments into the former limb territory [45–48]. Many studies have looked at the reorganization of the nervous system after amputation, but commonly in the perspective of phantom sensations (e.g., pain), which are likely result of ongoing neuroplasticity [49–52]. Further studies have shown the relevance of phantom sensations in relation to cortical plasticity. Strong relationships have been seen correlating phantom sensations and the degree of cortical reorganization [53, 54]. Further, approaches to manage phantom pain could serve to aid in sensory reorganization [55, 56]. As will be described in later sections, establishment of sensation from the prosthetic device likely plays a pivotal role in cortical plasticity that can affect motor learning processes.

Studies in animals have revealed mechanisms of complex plasticity that has a significant role in motor system physiology. Residual cortical limb representations may affect ongoing plasticity within cortical regions. Studies in adult rats with forelimb amputations after birth revealed that when forelimb stump representations in the contralateral somatosensory cortex was inactivated (or reduced [57]), principally hindlimb inputs were revealed. This suggests that forelimb stump representations can actively suppress the expansion of hindlimb inputs [58]. This seemingly proposes a similar mechanism thought to be involved in human studies using INB acutely [23]. Using positron emission tomography, GABA receptor binding was studied in unilateral amputees years following injury. Results showed increased GABA receptor binding in upper limb regions of the primary sensorimotor areas bilaterally, which suggests long-term stability of cortical reorganization of the stump and former limb territory [59].

**Fig. 2:**
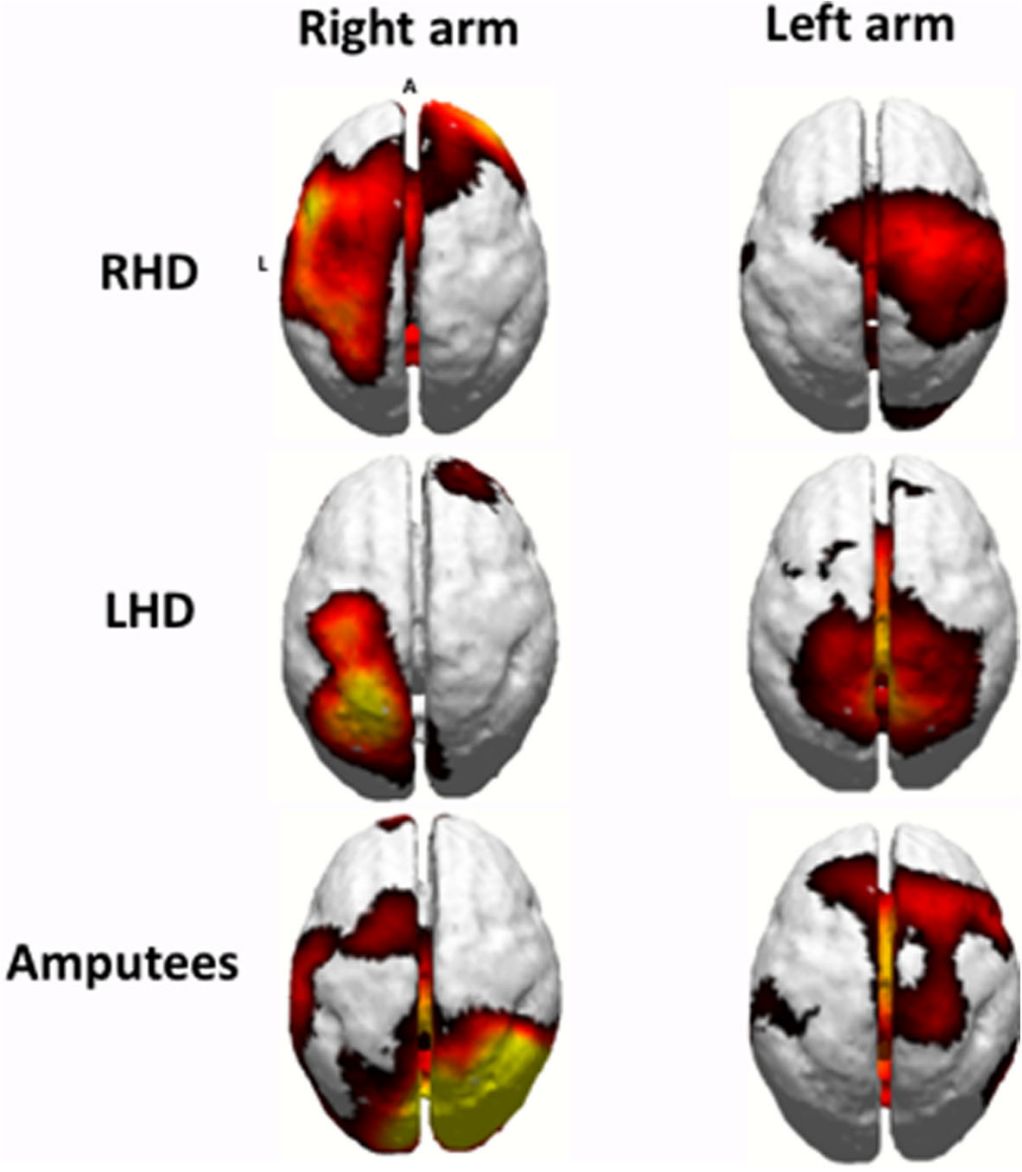
Current source density of beta band (18–22 Hz) activity during motor planning in each group (from [66], with permission from the publisher)

Recent studies report large-scale changes in neural networks including and beyond sensorimotor cortex following amputation [60]. This work takes advantage of functional connectivity of sensorimotor areas during resting state, avoiding confounds induced by specific motor tasks. The authors identified a progressive disconnection of the missing hand cortex and the sensorimotor network, with a concomitantly stronger connection with the so-called default mode network [61]. While it is unclear what is driving this network plasticity, it does reflect a functional shift of the entire sensorimotor network that could have rehabilitative consequences. In a case-study report, it was shown that amputees may recruit areas outside of the expected left parietofrontal system for motor-relevant tasks (attributed to the mirror neuron system), including the right temporoparietal junction (attributed to the mentalizing system [62]) [63]. This may correspond to clinical observations suggesting that amputees develop a behavioral adaptation of placing a greater emphasis on the visuomotor aspects of motor task performance [64, 65]. We have suggested that neural patterns seen in electroencephalography (EEG) reflect widespread changes in neural oscillations in amputees [66]. In this work, amputees performed a basic motor task with their amputated (right) arm, and then their sound limb to evaluate neuroplasticity that may occur following amputation. Effects were compared against a population of left- and right-handed persons with sound limbs. Results demonstrated that for movements with the sound limb amputees showed activation over the contralateral motor cortex that was greater than the persons with sound limbs. Movements with the amputated limb showed bilateral (and mainly right) parieto-occipital activation (Fig. 2). These examples help to illustrate the complex neural changes that follow in the cortex, which in the motor system may be influenced by changes in activation of projection areas to sensorimotor cortex [67]. However, argument can be made that cortical plasticity is a result of plasticity outside of the cortex.

#### Subcortical influences on cortical plasticity

Very few studies have investigated the role of subcortical reorganization on motor function related to upper limb loss. Studies in animal models have well demonstrated changes in deep brain structures that may impact cortical plasticity. These have focused on the sensory reorganization that can occur following limb loss by studying subcortical nuclei that relay ascending sensory information to higher-order areas. Of emphasis is to attempt to determine what subcortical areas might serve as a substrate for cortical reorganization. In the case of the upper limb, the posterior medial lemniscus pathway carries sensory information from the upper part of the body. Once peripheral axons enter the spinal cord, most branch to ascend into the dorsal columns and traverse into the medulla where a synapse is made on the cuneate nuclei. Relay neurons send projections to the ventral posterior nucleus of the thalamus, which sends information into the primary somatosensory cortex. Thus, possible brain regions for sensory cortical reorganization based on this projection may occur in the spinal cord, cuneate, thalamus, and/or intrinsic to the cortex. Studies of monkeys have demonstrated changes to motoneurons and spinal cord atrophy in the residual limb that could relate to cortical changes following amputation [68, 69]. Forelimb amputation in rats has demonstrated that over the course of 30 weeks post amputation, very little new sensory input is seen at the cuneate nucleus in the zone of former forepaw territory, and that the main reorganization occurs around the former forepaw zone [70]. This is despite the finding that shoulder input occupies the former forepaw barrel cortex within 4 weeks of amputation. This suggests that cuneate reorganization (including inputs into the cuneate [71]) is not related to cortical reorganization. Further studies revealed that cortical reorganization may be related to changes in ventral posterior lateral nucleus of thalamus (VLP), via latent subthreshold cuneothalamic residual limb inputs in the former limb territory [72]. This largely concurs with a prior study in neonatal forelimb removal in rats, showing that abnormal hindlimb receptive fields in the stump territory of primary sensory cortex is attributed to an increase in VPL neurons that have sensory receptive fields on the stump and hindlimb [73]. The resultant dual hindlimb-stump representation was not attributed to changes in intracortical connections within, or changes to thalamocortical projections to, somatosensory cortex. This suggests that cortical plasticity may have a thalamic origin, which, given the robust density of thalamocortical projections to motor cortex may suggest that if sensation is lost, thalamocortical plasticity may have profound impacts on cortical substrates for learning [73]. Studies have demonstrated that thalamocortical projections (notably from ventral anterior/ventral lateral thalamus (VA/VL) complex) into motor cortex have populations of neurons that show motor learning plasticity, by demonstrating biased activation towards learned tasks [74].

These studies identify the significant role that the thalamus (and thalamocortical pathways) play in shaping cortical plasticity after amputation, and suggest a role in motor learning. While it is presently unclear what role limb loss may have on VA/VL complex specifically, given widespread projections of these nuclei to frontal premotor areas [75], thalamocortical networks arising from VA/VL nuclei may have significant importance on how cortical networks involved in motor learning function following limb loss. While the VL thalamus sends efferents to primary motor cortex (ventral anterior nucleus of thalamus, principle segment; VLp), it also has dense connectivity to premotor (ventral anterior nucleus of thalamus, anterior segment; VLa) and posterior parietal (VL) cortices [76, 77]. Studying markers of glia cell activation, it was seen that increases in thalamic (but not sensorimotor cortical) glial signaling persisting years after nerve injury [78]. This finding further supports the hypothesis that thalamic pathways are vital to consider, and the role they play in functional plasticity long after injury. Further, these changes may differ based on the stage of development that amputation occurs [79]. While evidence is lacking specifically as to the nature of the role of VL in human neurophysiology and amputation, some relevance may be seen in studies detailing changes in beta-band oscillations at a thalamocortical network level. Physiological changes seen in cortical recordings demonstrating neuroplasticity using EEG [66] may be of thalamic origin due to the coherence between thalamus and cortex within specific frequencies of activation [80].

### Relevance of neuroplasticity to motor learning

What is the importance of cortical and subcortical neuroplasticity to motor learning in amputation? Regardless of the prosthesis device designs, motor learning is a vital component [81]. As previously discussed, significant technological advancements in prosthetics have yet to provide a device that can fully and immediately provide sensory establishment and motor control improvement without a practice-based learning/adaptation period. As described above, there is vast plasticity of thalamocortical circuits following amputation. It is possible that the development of prosthetic limb technology must consider how, or if, this underlying neuroplasticity is a challenge to prosthetic device learning and adaptation. There is evidence emerging in behavior and neurophysiology that underscores the significance of neuroplasticity and adaptation in motor learning for amputation. However, technology that relays sensory feedback from the prosthesis to the body appears not to be a requisite for motor control improvement.

#### Concepts with engineered prosthetic limb sensory feedback

Using brain machine interface approaches, it has been demonstrated that stable connectivity between primary cortex and the striatum is pivotal for skill learning with prostheses [82]. While this particular study focused on neuroprosthetics, the concept that there has to be stability between corticostriatal areas to provide a stable neural platform for motor learning is valuable. As we have reviewed, it seems evident that ascending sensory loss is capable of promoting cortical plasticity in motor cortex, the integrity of primary motor-striatal pathways in amputation becomes a pivotal issue in understanding neuroplastic changes that may impact prostheses that rely on neural signals. It is unclear what occurs in the striatum after amputation, but immobilization studies show a use-dependent function of motor activation. Immediately upon removing chronic immobilization in the upper limb, decreases in striatum activity is seen which can be recovered as motor control recovers [83]. Continued studies further isolated this effect to the putamen, showing that loss of putamen activation relates to loss of learned movement sequences during chronic immobilization [84]. These studies highlight the possibility that functional disuse can lead to maladaptive plasticity within the striatum, which could have negative consequences for motor learning. In the case of immobilization, removal of the orthotic gives rise to the natural limb and the pre-immobilization state. This return to the natural limb state is not possible in the case of amputation. Thus, understanding how prosthetic devices with sensory feedback may unmask this potential striatal-cortical maladaptive plasticity to encourage learning is a pivotal step in understanding prosthesis adaptation and motor learning.

Physiologically, research has demonstrated a critical role of proprioception in shaping motor cortex plasticity. Using transcranial magnetic stimulation, input-output curves and intracortical inhibition were measured before and after 10 h of limb immobilization [85]. Participants were placed in one of 3 groups, tactile vibration, proprioceptive vibration (yielding illusory movements), and a no-vibration control. Results showed that the absence of vibration caused a decrease in contralateral motor cortical excitability, but proprioceptive vibration (and to some degree tactile vibration) minimized this effect. Ipsilateral motor cortex showed hyperexcitability in the absence of vibration and tactile vibration groups, but did not show hyperexcitablilty under the proprioception vibration group. This result suggests that the absence of proprioception (and not just the loss of movement in immobilization) may cause hemispheric imbalance of excitability, which may pave the way for motor functional loss of the cortical motor representation. This suggests that ascending sensory feedback (through a cuneo-thalamo-cortical projection) can modify motor plasticity. This has strong relevance to amputation rehabilitation as it is unclear what the return of proprioception (via haptic feedback prosthetics) would do to potentially unmasking hemispheric imbalance seen chronic upper limb amputees [86, 87]. While motor learning may promote cortical excitability [88], studies have revealed that decreased excitability of primary cortex can suppress skill acquisition with the contralateral hand, while promoting learning with the ipsilateral hand [89]. Such a pattern could influence motor learning with prostheses without proprioceptive or haptic feedback. Behavioral evidence shows that in body-powered prosthesis users, motor learning and recall is significantly affected by the presence of proprioceptive motor errors (e.g., slips, drops) in a tactile visuomotor learning task [90]. In contrast, persons performing the task with sound limbs show reductions of error, and increased learning. This may suggest that proprioceptive feedback beyond haptic feedback is helpful to the learning process.

Sensation can be established with training using prostheses. Sensory representations of functional robotic hands can develop after 30 h of tactile feedback-based training on using motoric tasks with a robotic limb [91]. Evoked tactile sensation methods have demonstrated the possibility of evoking sensory perceptual thresholds as a finger map on the radial and ulnar sides of the residuum of forearm amputees, which could serve as a way to encode sensory feedback through training [92]. Targeted reinnervation approaches for prosthetics have demonstrated cortical remapping that suggests a return of sensorimotor control closer to their non-amputated state [93]. In a retrospective study, targeted reinnervation shows success in establishing sensory perception in the prosthetic limb, yet significant variability across persons exists [94]. These sensory based outcomes could be highly related to motor function improvements in persons with limb loss that are able to have sensory and motor reinnervation [95, 96]. As a recent example, advances have shown promise to implantable (and potentially wireless, [97]) electromyography systems that are capable of driving a prosthetic hand with upwards of 3° of freedom or isolated finger movements [98–100]. However, recent work evaluating motor unit activity revealed motor units that covered smaller surface areas (with high degree of overlap) and shorter action potential durations in amputees with targeted reinnervation compared to persons with sound limbs [101]. This could explain the difficulty in simultaneously coordinating >3° of freedom after targeted reinnervation [102], and stress the importance of a stronger understanding of motor unit physiology after reinnervation.

Exciting new work is highlighting the capacity to provide information about the intensity of sensory feedback capability to amputees with peripheral nerve electrodes [103]. However, as we will discuss below, rehabilitation paradigms to deal with the neuroplasticity from lost proprioception may also support motor learning with prostheses that do not have sensory feedback available. Indeed, it has been demonstrated that the introduction of vibrotactile feedback while training with prostheses has led to remarkable variability in motor outcomes including no benefit/maladaptive [104], modest benefit [105, 106], to significant benefit that was seen after prolonged training [107]. Such evidence should cause researchers to consider what the unique role of artificial sensory feedback might have on the nervous system, and whether improvements are driven by a training effect that may, or may not be, comparable with more rudimentary devices.

#### Concepts without engineered prosthetic limb sensory feedback

Restoring sensation through advanced prostheses that intentionally provide sensory feedback may not be the only option. Indeed, sensation can be acquired through body powered devices, as position and vibration of the cabling support provides position and force information [108]. This mechanical information can be harnessed to support object identification [109]. This may relate to how non-biological implements (such as tools) can obtain a sensorimotor embodiment after training [110]. In this case, neurons of the ventral premotor cortex of the monkey that fired to fingers additionally fired to seen actions of learned pliers. It is possible that sensory feedback obtained during training of the pliers afforded this process. In the case of amputation, motor “entrainment” without direct sensation has been seen in the so called “rubber hand illusion” [111, 112] where stimulation of a rubber hand activates parietofrontal areas in observers that have developed stimulus synchrony with the rubber hand [113–115]. Participants will even report a sense of ownership with the rubber hand [116], and can develop identity with a supernumerary hand without a loss of ownership of their own hand [117]. Evidence of identity with artificial hands, even those that reshape the human body (e.g., supernumerary hands), is critical to consider as it suggests that ownership is not essentially tied to the actual presence of ascending sensory feedback (i.e., feedback delivered through an engineered haptic feedback system in the rubber hand/prosthesis) but merely the ability to translate non-biological componentry into body schema that may involve some level of learned sensory assimilation.

This opens the possibility that training paradigms may sufficiently enable sensorimotor embodiment of prostheses for motor control with [91] or without sensory feedback from a prosthetic device. In an early neural study [118], we sought to identify if sensorimotor areas might be sensitive to various approaches to motor learning. Chiefly in the case of amputation, learning motor skills with prostheses may be difficult to learn from persons with sound limbs, compared to learning with another prosthesis user based on differences in limb state between the observer (amputee) and the observed (trainer). Persons with sound limbs and traumatic upper limb amputee prosthesis users imitated actions of other prosthesis users or intact limbs. All of the amputees were “low users” of prostheses, indicated by average time device was worn per day (~4.4 h/day) and self-report. Results showed that when amputees imitate intact subjects, they have a persistent bilateral occipito-parietal and right temporoparietal positivity in motor planning. However, when amputees emulated other amputees, they showed an emergent left parietal and mesial frontal activation during motor planning. The consequence of right hemispheric activation in the amputation group might reflect heightened visuospatial needs in learning to translate action of the sound limb into appropriate motor outcomes (as demonstrated in tool use in healthy adults [119–122]). It is known that amputees face increased difficulty in motor imagery [123], which may affect interfere with developing motor skills in rehabilitation. This is would be critical barrier to skill acquisition and would demonstrate that dissimilarity of limb state is a challenge to encoding action skills in a sensorimotor reference frame in observational rehabilitation. It is also possible that non-invasive brain stimulation may also help support entrainment of motor patterns to help support motor learning [124], with potential applications to using prostheses [125]. This could be of strong benefit to support training to establish proper neural circuits to improve motor skill coordination with prostheses.

Based on our prior work [118], we have proposed a possible neural adaptation modeled in persons with sound limbs relevant to observation-based rehabilitation training. We utilized a fictive model of amputation [126] to longitudinally assess neurobehavioral adaptations to learning prosthesis use based on learning with a prosthesis user (“matched limb”) versus a person with sound limbs (“mismatched limb”). Immediate to fitting with the prosthesis, and on the first day of training, persons in the matched limb group showed dominant left parietofrontal activation patterns. However, persons in the mismatched group showed heightened occipital and parietal activity bilaterally, with decreased mesial premotor/motor activity. These patterns held stable through training. This suggests that proper training may promote sensorimotor activation patterns for prosthesis use, which may better enable functional adaptation to occur. We have identified similar findings in kinematics, demonstrating improved motor control outcomes with matched limb training [126, 127].

### Other relevant approaches

Research is also emphasizing training concepts that could be relevant to amputees prior to prosthesis fitting. Studies have proposed the idea of bilateral transfer of motor skills across limbs using a prosthesis simulator [128]. The motivation of this idea is to take advantage of learning that can be done in the unilateral amputee’s sound limb that may promote motor skill learning in the weeks to months before they can be fit with a prosthetic limb. For one example, therapies that focus on interlimb transfer and training of skilled use of the residual limb for future prosthesis usage [129, 130] may be advantageous to start training directly after amputation and promote prosthesis-based motor plans in the affected cortex. Research has shown that the “deprived cortex” (contralateral to the amputation) is used by whichever limb individuals are using most, suggesting mechanisms of experience-driven plasticity shaping bilateral sensorimotor reorganization [131]. Such experience-dependent plasticity may be harnessed in motor learning. This is valuable, as training that promotes use of the residual limb should be of emphasis. It may be that such training focus could do well in limiting putative maladaptive cortical and subcortical plasticity. In one example, the use of virtual reality training may enhance affected limb use before fitting to optimize outcomes. While this concept has been approached with advanced prostheses [132], it may also extend to all forms of devices.

Pharmacological interventions in amputees may influence motor control and motor learning. For example, phantom limb pain has shown resolution with duloxetine (serotonin-norepinephrine reuptake inhibitor) and pregabalin (increases GABA production) [133]. It has been noted that duloxetine may have benefits to psychomotor performance [134]. However, it is argued that decreases in local GABA are beneficial to motor learning related increases in activity in primary motor cortex [135] while increases can suppress motor learning [24] which may suggest a negative effect of pregabalin. Pain control after amputation can involve prolonged use of many pharmacological agents including anticonvulsants, tricyclic antidepressants, and even electrical nerve stimulation [136]. An improved understanding of the role these agents may play in motor learning and neuroplasticity are vital in developing empirical evidence in future studies, particular in animal studies where neuropharmacology can be better evaluated at a cellular and systems level.

## Conclusions

The complexity of neural changes following amputation that may have profound impacts on motor planning, execution and learning offer unique opportunities to better inform rehabilitation strategies in the future. Understanding therapies and technology through the lens of whether these approaches can unmask or utilize ongoing neuroplasticity has great value. Notably, evidence that neuroplasticity, “embodiment” and motor learning may develop without restorative sensory feedback from prostheses helps illustrate the complex neuroanatomy and physiology that can contribute to challenges related to upper limb loss. Training and practice are a core aspect of all motor improvements seen with advanced bionic and more rudimentary prostheses. Specifically, these observations should drive the neuroscience and neuroengineering community to collaboratively focus more on what factors might enhance plasticity to restore function, and whether various forms of sensory feedback provide common or distinct mechanisms to improved function, neural plasticity and adaptation. This type of evidence is critical to demonstrating the unique capacities for certain technologies in restoring function, and provide a richer literature to support clinical integration. As well, while not specifically covered in this review, a vital concept is understanding how control interfaces (body bowered versus myoelectric), physical factors such as limb length, surgical methods and socket design (particularly important for myoelectric designs) augment neural adaptations and influence motor learning [3, 41, 137–139]. Further, the fundamentals of motor control in amputees (e.g., functional lateralization of the sound limb or the affected limb with prostheses, how sensory feedback is used in affected and unaffected limbs) remains enigmatic. Understanding how these concepts work with device designs and emerging technology (such as sensory-based prostheses and even supernumerary limbs) can enable a stronger integration of neuroscience, neuroengineering, and clinical research (as has been well implemented in other rehabilitation models, such as stroke [140]) to yield stronger scientific and technical knowledge to improve outcomes. In discussing evidence-based approaches to prosthetic rehabilitation [12], the authors well state the challenges of integrating fast-moving technological designs into the clinic based on evidence that can be difficult to interpret, and lacking in rigor for health-care financers. This is a fundamental challenge. While “research” (in [12], this more strongly, but not exclusively means technological development) is needed to offer solutions on device designs, it is possible that collaborative neuroscience and neurorehabilitation approaches may shape both training methodology and technological development. Answers to questions about the necessity of sensory feedback, motor learning with prostheses, neuroplasticity, and needs of the patient mutually inform each other to shape a deeper and relevant knowledge base for rehabilitation. Thus, a neuroscience, neuroengineering, and clinical collaborative/informative model will likely help support a stronger future for shaping positive rehabilitation outcomes. This is essentially done by building collaborations across these communities dedicated to evidence-informed practice that can become the basis for fundamental changes in rehabilitation.

## Abbreviations

EEG: Electroencephalogaphy
fMRI: Functional magnetic resonance imaging
GABA: Gamma-aminobutyric acid
INB: Ischemic nerve block
VA: Ventral anterior nucleus of thalamus
VAa: Ventral anterior nucleus of thalamus, anterior segment
VAp: Ventral anterior nucleus of thalamus, principle segment
VL: Ventral lateral nucleus of thalamus
VLP: Ventral posterior lateral nucleus of thalamus
